# Targeting Conserved Pathways in 3D Spheroid Formation of Diverse Cell Types for Translational Application: Enhanced Functional and Antioxidant Capacity

**DOI:** 10.3390/cells12162050

**Published:** 2023-08-11

**Authors:** Chia-Chi Chang, Shih-Sheng Jiang, Fang-Yu Tsai, Pei-Ju Hsu, Chen-Chan Hsieh, Li-Tzu Wang, Men-Luh Yen, B. Linju Yen

**Affiliations:** 1 Graduate Institute of Life Sciences, National Defense Medical Center (NDMC), Taipei 114, Taiwan; 2 Regenerative Medicine Research Group, Institute of Cellular & System Medicine, National Health Research Institutes (NHRI), Zhunan 350, Taiwan; 3 National Institute of Cancer Research, NHRI, Zhunan 350, Taiwan; 4 Department of Obstetrics/Gynecology, National Taiwan University (NTU) Hospital & College of Medicine, Taipei 100, Taiwan

**Keywords:** three-dimensional (3D) spheroid formation, pluripotent stem cells, mesenchymal stem cells, cancer cells, cholesterol synthesis, cytoskeleton modification, antioxidant capacity

## Abstract

Three-dimensional (3D) in vitro spheroid/organoid culture increasingly appears to better mimic physiological states than standard 2D systems. The biological consequence of 3D spheroids, however, differs for different cell types: for pluripotent embryonic stem cells (ESCs), differentiation and loss of stemness occur, while the converse is true for somatic and cancer cells. Despite such diverse consequences, there are likely conserved mechanisms governing 3D spheroid formation across cell types that are unknown but could be efficiently targeted for translational application. To elucidate such processes, we performed transcriptome analysis with functional validation on 2D- and 3D-cultured mouse ESCs, mesenchymal stromal/stem cells (MSCs), and cancer cells. At both the transcriptomic and functional levels, 3D spheroid formation resulted in commitment towards known cell-specific functional outcomes. Surprisingly in all cell types, downregulation of the cholesterol synthesis pathway was found during 3D spheroid formation, with modulation concomitantly affecting 3D spheroid formation and cell-specific consequences; similar results were seen with human cell types. Furthermore, improved antioxidant capacity after 3D spheroid formation across cell types was further enhanced with modulation of the pathway. These findings demonstrate the profound cell-specific consequences and the translational value of understanding conserved mechanisms across diverse cell types after 3D spheroid formation.

## 1. Introduction

In vitro mammalian cell culture is an indispensable tool for dissection of normal and pathological biological processes and mechanisms. As invaluable as the traditional 2-dimensional (2D) in vitro cell culture has been, this system has many limitations including the inability to mimic cell–cell and cell–extracellular matrix (ECM) interactions or the metabolic processes of cells within organs/tissues which are 3-dimensional (3D) in nature [1,2]. In fact, many studies have demonstrated that 2D in vitro cell cultures alter the cultured cell at both the genetic and epigenetic level, leading to genetic/chromosomal aberrations and functional phenotypes that drift away from its original state [3,4]. Thus, while 2D in vitro cell culture is convenient and cost-effective, its physiological relevance is increasingly being questioned. Emerging evidence demonstrates that 3-dimensional (3D) in vitro culture conditions may better reflect in vivo physiologic conditions [5,6,7]. Three-dimensional cell culture was first used to elucidate pluripotent stem cell (PSC) differentiation capacity and mechanisms [8,9] as well as to select for somatic stem cell populations, particularly for neural and mammary stem cells [10,11]. More recently, the ability to form spheroids in 3D culture conditions has been used to identify cancer-initiating cells or cancer stem cells [12,13]. As important as 3D in vitro culture systems has become, there is surprisingly scant information on uniformed pathways governing this capacity to retain viability and form spheroid structures in 3D non-adherent conditions among diverse 2D plastic-adherent cell types; such knowledge may be critical to further the understanding of functional outcomes which can likely improve therapeutic applications. Given the divergent biological consequences of 3D spheroid formation in different stem cell and somatic cell types, we were interested in finding unified mechanisms in regulation of 3D spheroid formation across highly diverse cell types including pluripotent stem cells, somatic stem cells, and cancer cells. Transcriptome analyses of 2D- and 3D-cultured mouse embryonic stem cells (ESCs), mesenchymal stem cells (MSCs), and cancer cells revealed that the cholesterol synthesis pathway was uniformly downregulated in all three cell types after 3D spheroid formation. Functional validation demonstrated that modulation of this pathway controlled 3D spheroid formation in murine as well as similar human cell types. Moreover, modulation of the cholesterol synthesis pathway modulated the functional outcome specific to each cell type and enhanced the stress response in all assayed cell types. Our findings, therefore, can be extrapolated to diverse stem cell and somatic cell types and likely have implications in improving the use of 3D in vitro culture systems to therapeutic use.

## 2. Materials and Methods

### 2.1. Cell Culture

All cell lines were obtained from American Type Culture Collection (ATCC; Manassas, VA, USA) and cultured as indicated by the manufacturer; all cell culture reagents were purchased from Thermo Fisher (Waltham, MA, USA) with the exception of leukemia inhibitory factor (LIF) (MilliporeSigma, Burlington, MA, USA) and mTeSR™1 (StemCell technologies, Vancouver, BC, Canada). Mouse ESCs (mESCs; CRL-1934) were cultured in DMEM-high glucose, 10% fetal bovine serum (FBS), 10^3^ U/mL LIF, 100 µM non-essential amino acids (NEAA), 1 mM sodium pyruvate, 2 mM L-glutamine, 75 μM β-mercaptoethanol, and 100 U/mL antibiotics. Murine MSCs, M210B4 (CRL-1972), were cultured in RPMI-1640, 10% FBS, and 100 U/mL antibiotics. Murine lung adenocarcinoma Lewis lung cells (LLCs; CRL-1642) were cultured in DMEM-high glucose, 10% FBS, and 100 U/mL antibiotics. Human iPSCs obtained from the Bioresource Collection and Research Center (BCRC; Hsinchu, Taiwan) were generated by Sendai Virus induction with all four Yamanaka factors into human peripheral blood mononuclear cells [14] and cultured in mTeSR™1. As previously described, placenta-derived MSCs (P-MSCs) were isolated from term human placenta tissue of healthy donors after obtaining informed consent, approved by the Institutional Review Board according to the Declaration of Helsinki guidelines [15], and cultured in standard MSC medium of DMEM-low glucose, 10% FBS, 2 mM L-glutamine, and 100 U/mL antibiotics [16]. The human lung adenocarcinoma cell line SK-MES-1 was cultured in DMEM-high glucose, 10% FBS, and 100 U/mL antibiotics. All human cells were handled following institutional biosafety guidelines.

### 2.2. Three-Dimensional Spheroid Formation

Three-dimensional spheroid formation was performed as previously reported [9,17,18]. Briefly, culture dishes (10 cm) were filled with 7 mL of 1.6% agarose (Focus Bioscience, Murarrie, QLD, Australia) dissolved in phosphate buffered saline (PBS) to prevent cell adhesion, and with seeding of 10^6^ cells per dish. Cell viability was detected with the Live/Dead staining assay (Thermo Fisher) and visually determined with confocal microscopy (Leica Microsystems, Wetzlar, Germany) [19]. Mevastatin, methyl-β-cyclodextrin (MβCD), cholesterol, blebbistatin, and cytochalasin D are all from MilliporeSigma. For inhibition, depletion, and repletion of cholesterol synthesis, mevastatin (10 mM), MβCD (150 mM), and cholesterol (30 mM) were used, respectively, according to previous reports [20,21]. For myosin II and F-actin polymerization inhibition, blebbistatin (10 mM) and cytochalasin D (2 mM) were used, respectively, according to previous reports [22,23]. Cells were cultured in 3D culture conditions and treated with the agents at the indicated concentrations (listed on Table S1) for 72 h. For H_2_O_2_ treatment, cells were treated using the same cell culture process described above, then 2D plastic-adherent cells and formed 3D spheroid would change cultured medium of each cell type with 500nm of H_2_O_2_ for 2 h [19]. Three-dimensional spheroids were visualized using a phase-contrast microscope (Olympus, Tokyo, Japan) and analyzed by ImageJ (version 1.53j, National Institutes of Health, Bethesda, MD, USA) [24]. Quantification was performed as previously reported [25,26] with average spheroid area obtained from counts using 9 fields for each experimental condition.

### 2.3. Cell Cycle Analysis

Cell cycle analysis was performed as previously reported [16]. Briefly, cells were harvested by HyQTase (Thermo Fisher) and washed with PBS. The pellets were then fixed in 70% ethanol for 1 h at room temperature, followed by incubation in 0.5 mL staining solution (0.1% Triton X-100 in PBS, 100 m g/mL RNase A, and 50 m g/mL propidium iodide (all from MilliporeSigma) at 37 °C for 20 min. The cell cycle distribution was analyzed by using FACSCalibur and drawn using CellQuest software (version 5.1, BD Biosciences, San Jose, CA, USA).

### 2.4. Gene Expression Microarray Experiments and Bioinformatics Analyses

Total RNA was isolated using RNeasy Mini Kits (Qiagen, Hilden, Germany), and the samples with A260/280 greater than 1.9 were then applied to reverse transcription. Gene expression profiles of obtained cDNA were examined with mouse Gene 2.0 ST array (Affymetrix, Santa Clara, CA, USA) performed by the core facility of the National Health Research Institutes (NHRI; Miaoli, Taiwan) according to standard operation procedures [27]. The raw intensities were processed by signal space transformation-robust multiple-array average (SST-RMA) normalization method. Fold-change calculations and principal component analysis (PCA) were performed by Partek Genomics Suite software (Chesterfield, MO, USA). Comparison of enriched pathways/biological processes were performed using gene set enrichment analysis (GSEA) [28], and Metascape [29]. The gene interaction networks were generated by Ingenuity Pathway Analysis (IPA; Qiagen). Microarray data generated in this study have been deposited to the NCBI GEO database. The accession number is GSE99942 (https://www.ncbi.nlm.nih.gov/geo/query/acc.cgi?acc=GSE99942, accessed on 12 June 2017). Transcriptome data of human iPSCs (GSE116574), MSCs (GSE174619), and lung cancer (GSE142616) were obtained from a public database.

### 2.5. Real-Time RT-PCR (qPCR)

qPCR was performed as we previously reported [16]. Briefly, total RNA was extracted using TRI reagent (MilliporeSigma), quantified with NanoDrop spectrophotometer (Nyxor Biotech, Paris, France) and converted to cDNA by using RevertAid H Minus reverse transcriptase (Thermo Fisher). qPCR was performed using ABI PRISM 7500 system (Applied Biosystem, Foster City, CA, USA) with SYBR Fast qPCR kit (MilliporeSigma). Primer sequences are listed in Table S2. All reactions were performed in triplicate and normalized to glyceraldehyde 3-phosphate dehydrogenase (Gapdh).

### 2.6. Immunofluorescent Staining

Immunofluorescent staining for F-actin polymerization was performed as previously reported [30]. Spheroids were fixed with 4% paraformaldehyde (MilliporeSigma) for 10 min and permeabilized with 0.5% Triton-X 100 in PBS overnight. Cells were stained with rhodamine phalloidin (1:200; Thermo Fisher) and counterstained with 4′,6-diamidino-2-phenylindole (DAPI, 1:10,000; Thermo Fisher) for 3 h at room temperature. Staining was visualized using a confocal microscope (Leica TCS SP5). The immunofluorescent intensity of F-actin was analyzed by ImageJ.

### 2.7. Measurement of Cell Growth

Cell proliferation was determined by the MTT assay ((3-4,5-dimethylthiazol-2-yl)-2,5-diphenyltetrazolium bromide; MilliporeSigma) according to manufacturer’s instructions. Cells were plated into 24-well plates at an initial density of 10,000 cells/well. After attachment, cells were treated with mevastatin at the indicated concentration for 24 h. After treatment, cells were incubated with 500 μg/mL MTT for 2 h. Blue formazan was dissolved in DMSO, and the absorbance values were measured at 570 nm with a SpectraMAX 250 microplate reader (Molecular Devices, Sunnyvale, CA, USA). Cell viability was normalized as a percentage, compared to control wells.

### 2.8. Statistical Analysis

For comparisons between 2 groups (i.e., 3D vs. 2D), statistical analysis was performed with Student’s *t* tests. For comparisons of multiple conditions (i.e., 3D spheroid formation, qPCR, cell growth, cell cycle, and immunofluorescent staining), statistical analysis was performed with one-way ANOVAs. Results are expressed as mean ± SEM, and a *p*-value of <0.05 was set for significance.

## 3. Results

### 3.1. Diverse Cell Types Including ESCs, MSCs, and Cancer Cells All Have Spheroid-Forming Capacity in 3D In Vitro Culture

**Figure 1:**
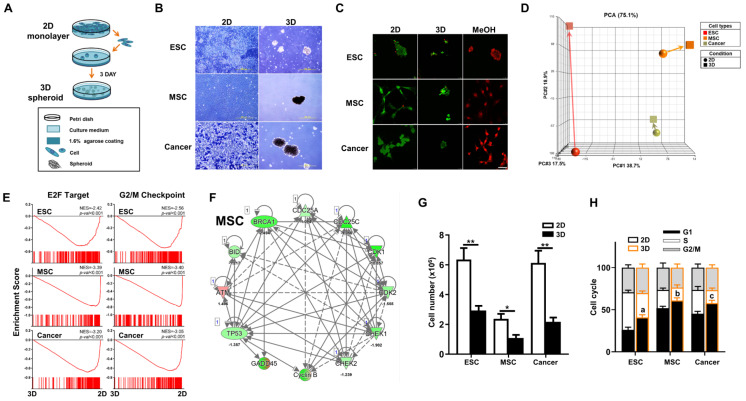
Diverse cell types including mouse embryonic stem cells (ESCs), mesenchymal stromal/stem cells (MSCs), and cancer cells all have spheroid-forming capacity in 3D in vitro culture. (**A**) Schematic diagram of 3D spheroid culture method. A total of 10^6^ cells of ESCs, MSCs, or Lewis lung cancer cells (cancer) were cultured in 3D conditions for 3 days to induce spheroid formation. Two-dimensional culture was performed according to standard practice (please see Section 2). (**B**) Morphology of ESCs, MSCs, and cancer cultured for 3 days in 3D spheroid vs. 2D conditions as visualized by phase-contrast microscopy. Scale bar, 500 µm. (**C**) Live/dead staining with Calcein acetoxymethyl ester (Calcein-AM, Green, living cells) and ethdium homodimer-1 (EthD-1, Red, dead cells) of ESCs, MSCs, and cancer cells cultured for 3 days in the 3D spheroid vs. 2D condition. Diverse cell types of the 3D condition would be treated with methanol for 30 min as dead positive control. Scale bar, 50 µm. (**D**) Principal component analysis (PCA) of transcriptome profiles of ESCs, MSCs, or cancer cells cultured in 2D or 3D conditions. (**E**) Bioinformatics analyses of transcriptomic profiles of 3D vs. 2D on the three cell types using GSEA. Significant enrichment and downregulation of cell cycle E2F (G1 phase) and G2/M gene sets in ESCs, MSCs, and cancer cells cultured in 3D vs. 2D conditions. (**F**) Ingenuity Pathway Analysis (IPA)—based pathway analysis for cell cycle related genes in transcriptomic profiles of MSCs cultured in 3D vs. 2D conditions. Upregulated genes are indicated in red and downregulated genes in green. Solid and dashed lines indicate direct and indirect interactions, respectively. Results for 3D- vs. 2D-culture ESCs and cancer cells are shown in Figure S1. (**G**) Cell numbers of ESCs, MSCs, and cancer cells cultured in 3D vs. 2D conditions. Data are presented as mean ± SEM. *, *p* < 0.05 and **, *p* < 0.01, compared with 2D condition. (**H**) Cell cycle dynamics of ESCs, MSCs, and cancer cells cultured in 3D vs. 2D conditions as analyzed by flow cytometry. Data are presented as mean ± SEM. a, *p* < 0.01, for comparison of G1 phase in 3D- vs. 2D-cultured ESCs; b, *p* < 0.05, for comparison of G1 phase in 3D- vs. 2D-cultured MSCs; and c, *p* < 0.05, for comparison of G1 phase in 3D- vs. 2D-cultured cancer cells.

In 3D culture conditions (Figure 1A), we found that mouse ESCs, the MSC line M210B4 (MSCs), and the LLC cell line (lung cancer cells), one of the best published murine cancer cell lines, are all capable of spheroid formation (Figure 1B). To confirm cell viability after 3D spheroid formation, we performed live/dead staining and found negligible cell death for all three cell types cultured in either 2D or 3D conditions (Figure 1C). To elucidate the biological outcome of 3D spheroid formation in diverse cell types, we performed transcriptome analyses on these three different cell types in 2D and 3D culture conditions. Principal component analysis (PCA) of the transcriptomes of 2D- and 3D-cultured cells revealed that in vitro culture conditions induced a more profound overall gene expression shift in ESCs than the other two cell types (Figure 1D). Previous studies demonstrate that 3D culture of many types of cells result in cell cycle arrest [13,31,32,33]. Our transcriptome analyses of 3D vs. 2D via gene set enrichment analysis (GSEA) revealed highly significant enrichment and downregulation of cell cycle pathways, E2F target (G1 phase) and G2/M checkpoint, in all three cell types after 3D culture conditions (Figure 1E). Similarly, Ingenuity Pathway Analysis (IPA)-based pathway analysis also demonstrated that cell-cycle-regulated genes were significantly downregulated in all three cell types after 3D spheroid formation (Figure 1F and Figure S1). To validate the pathway analysis, cell counting demonstrated significant decreases in cell number after spheroid formation in all three cell types (Figure 1G). Further validation using flow cytometry analysis for cell cycle dynamics significantly increases in G1 cell cycle arrest and decreases in S phase in all three cell types after culturing in 3D conditions (Figure 1H). Taken together, downregulation of the cell cycle and decreased cell proliferation was seen after 3D spheroid formation across diverse cell types.

### 3.2. Three-Dimensional Spheroids of Diverse Cell Types Demonstrate Distinct and Biologically Relevant Transcriptomic Changes

**Figure 2:**
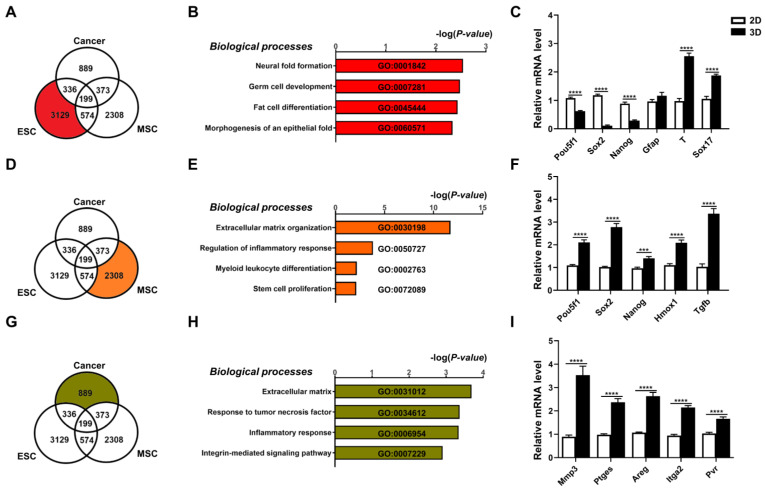
Three-dimensional spheroids of diverse cell types demonstrate distinct and biologically relevant transcriptomic changes. Transcriptome analyses of differentially expressed genes (DEGs; cutoff > 1.7-fold difference) of all three cell types cultured in 3D vs. 2D conditions visualized as a Venn diagram for (**A**) ESCs; (**B**) bar graph of the Gene Ontology (GO) terms enriched in these DEGs; (**C**) real-time PCR (qPCR) validation for gene expression levels of pluripotency genes (Pou5f1/Oct4, Sox2, Nanog) and three germ layer genes (ectoderm: glial fibrillary acidic protein, Gfap; mesoderm: T-Box Transcription Factor T, Tbxt/T; and endoderm: Sox17). For MSCs, (**D**) Venn diagram visualization of DEGs from transcriptome analyses of 3D vs. 2D conditions; (**E**) bar graph of GO terms enriched in these DEGs; and (**F**) qPCR validation for gene expression levels of pluripotency genes (Pou5f1, Sox2, Nanog), immune- and inflammation-related genes (heme oxygenase 1, Hmox1; and transforming growth factor beta 1, Tgfb1) in MSCs cultured in 3D vs. 2D conditions. For cancer cells, (**G**) Venn diagram visualization of DEGs from transcriptome analyses of 3D vs. 2D conditions; (**H**) bar graph of GO terms enriched in these DEGs; and (**I**) qPCR validation for gene expression levels of ECM modification enzyme (matrix metalloproteinase-3, Mmp3), integrin molecules (integrin alpha-2, Itga2; and poliovirus receptor/CD155, Pvr), and inflammation-response factors (prostaglandin E synthase, Ptges; and amphiregulin, Areg) in cancer cells cultured in 3D vs. 2D conditions. Gapdh, internal control. Data are presented as mean ± SEM. ***, *p* < 0.001, and ****, *p* < 0.0001, compared with 2D-cultured condition.

To assess specific transcriptome level changes in each cell type brought about by 3D culture conditions, we analyzed changes in gene expression induced by culturing in 3D vs. 2D conditions. Using a cut-off value of 1.7-fold change, a combined total of 7808 genes were found to be differentially expressed genes (DEGs). Of these genes, 3129 DEGs were specific for 3D- vs. 2D-cultured ESCs (Figure 2A), and analyses of these DEGs using Metascape revealed highly significant enrichment of lineage differentiation pathways, e.g., neural fold formation, morphogenesis of epithelial fold, germ, and fat cell differentiation (Figure 2B). Verification with qPCR analysis demonstrated decreases in expression of pluripotency factors Pou5f1/Oct4, Sox2, and Nanog and increases in expression of markers of germ layers (T-Box Transcription Factor T, Tbxt/T for mesoderm; and Sox17 for endoderm) (Figure 2C). These results indicate that in 3D conditions ESCs recapitulated developmental processes, resulting in differentiation with loss of pluripotency/stemness [34]. For MSCs, 2308 genes were differentially expressed in 3D vs. 2D conditions (Figure 2D), and analyses of these DEGs revealed significant enrichment of processes related to stem cell proliferation, extracellular matrix (ECM), immune and inflammation modulation capacities (Figure 2E). qPCR verification demonstrated significant upregulation of pluripotency genes (Pou5f1/Oct4, Sox2, and Nanog) and immunomodulatory genes (heme oxygenase 1, Hmox1; and transforming growth factor beta 1, Tgfb1) in MSCs after 3D culture (Figure 2F) as has been previously reported [35,36]. For cancer cells, 889 genes were differentially expressed in 3D vs. 2D conditions (Figure 2G), and analyses of these DEGs revealed significant enrichment of processes related to ECM, integrins and inflammation (Figure 2H). qPCR verification demonstrated significant upregulation of the ECM modification enzyme matrix metalloproteinase-3 (Mmp3), the inflammation response factors prostaglandin E synthase (Ptges) and amphiregulin (Areg), and the integrin molecules (integrin alpha-2, Itga2 and poliovirus receptor/CD155, Pvr) in cancer cells after 3D spheroid formation (Figure 2I) as has been previously reported [12,13]. Collectively, DEG transcriptomic results specific for each of the three different stem cell/cell types demonstrated that 3D culture conditions can recapitulate distinct cell-specific functional outcomes.

### 3.3. Downregulation of Cholesterol Synthesis Pathway Significantly Enhanced 3D Spheroid Formation of Diverse Cell Types

**Figure 3:**
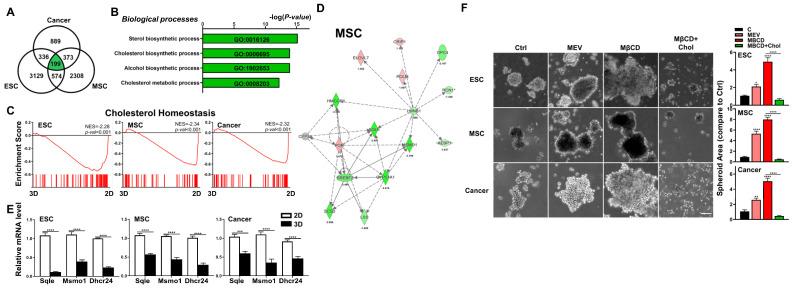
Downregulation of cholesterol synthesis pathway significantly enhanced 3D spheroid formation of diverse cell types. (**A**) Venn diagram showing overlap between DEGs (cutoff > 1.7-fold difference) in 3D vs. 2D conditions for each cell type. (**B**) GO terms enriched among DEGs of 3D vs. 2D conditions for each cell type. (**C**) Significant enrichment and downregulation of the cholesterol synthesis pathway genes via GSEA in ESCs, MSCs, and cancer cells cultured in 3D vs. 2D conditions. (**D**) IPA-based pathway analysis for the cholesterol synthesis pathway of 3D- vs. 2D-cultured MSCs. Upregulated genes are indicated in red and downregulated genes in green. Solid and dashed lines indicate direct and indirect interactions, respectively. Genes in gray are generated based on IPA and not from transcriptome data. Results and individual gene expression levels for 3D- vs. 2D-cultured ESCs and cancer cells are shown in Figure S2. (**E**) qPCR analysis for expression levels of cholesterol synthesis enzymes 24-dehydrocholesterol reductase (Dhcr24), methylsterol monooxygenase 1 (Msmo1), and squalene epoxidase (Sqle) in ESCs, MSCs, and cancer cells cultured in 2D and 3D conditions; Gapdh, internal control. Data are presented as mean ± SEM. ***, *p* < 0.001, and ****, *p* < 0.0001, compared with 2D cultured conditions. (**F**) Phase contrast microscopy of 3D-cultured ESCs, MSCs, and cancer cells treated with the cholesterol synthesis inhibitor mevastatin (Mev; 1 μM), depletion of cholesterol by methyl-β-cyclodextrin (MβCD; 1.5 mM), or addition of cholesterol (1.5 mM MβCD + 100 μg/mL cholesterol condition). Scale bar, 100 μm. Bar figures represent quantification of area of 3D spheroids. Data are presented as mean ± SEM. *, *p* < 0.05, **, *p* < 0.01, and ****, *p* < 0.0001 compared to 3D control or between conditions as indicated by bar.

To find uniformly modulated pathways involved in 2D culture to 3D spheroid formation in these three diverse cell types, we looked for similarly modulated pathways within the DEGs and found 199 genes which fit these criteria (Figure 3A). Further analyses using either Metascape or GSEA surprisingly revealed negative enrichment for the cholesterol synthesis pathway in all three cell types after 3D spheroid formation (Figure 3B,C). IPA further demonstrated in all three cell types after 3D spheroid formation, gene-networks associated with cholesterol synthesis were consistently the most downregulated (Figure 3D and Figure S2). To verify the bioinformatics findings, we performed qPCR analysis in all three cell types cultured in 2D and 3D conditions for expression level changes in key genes encoding enzymes involved in cholesterol synthesis including 24-dehydrocholesterol reductase (Dhcr24), methylsterol monooxygenase 1 (Msmo1), and squalene epoxidase (Sqle), and found that in all three cell types after 3D culture, significant downregulation of all analyzed genes occurred (Figure 3E). To functionally validate the transcriptomic results, we performed 3D culture of these three cell types with various modulators of the cholesterol pathway including mevastatin, an inhibitor of cholesterol synthesis [37], or methyl-β-cyclodextrin (MβCD), which depletes cell membrane cholesterol when given alone [38]. In 3D culture, we found that inhibition of cholesterol synthesis with mevastatin at non-cytotoxic doses, or depletion of cholesterol using MβCD alone significantly increased spheroid formation capacity in all three murine cell types (Figure 3F; dose–response cytotoxicity information for mevastatin in Figure S3). Conversely, when cholesterol levels are increased with the use of MβCD in conjunction with exogenous cholesterol [38], significantly decreased 3D spheroid formation capacity was seen in all three murine cell types (Figure 3F). Our data therefore demonstrate that the cholesterol pathway is critical in 3D culture-mediated spheroid formation of diverse cell types.

### 3.4. Inhibition or Depletion of Cholesterol Enhances 3D Spheroid Formation across Diverse Cell Types via Myosin Contraction and F-Actin Stabilization

**Figure 4:**
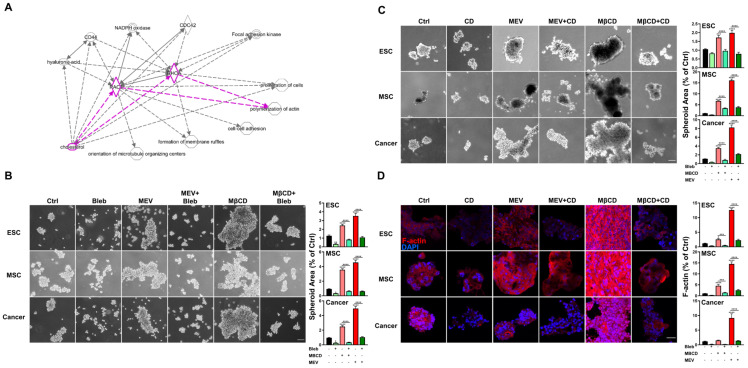
Inhibition or depletion of cholesterol enhances 3D spheroid formation across diverse cell types via myosin contraction and F-Actin stabilization. (**A**) IPA-predicted analysis for cholesterol-associated cytoskeleton modification including F-actin polymerization (highlighted). Solid and dashed lines indicate direct and indirect interactions, respectively. (**B**) Phase contrast microscopy of spheroid formation using ESCs, MSCs, and cancer cells in 3D conditions with or without Mev (1 μM), MβCD (1.5 mM), and blebbistatin (Bleb; 1 μM), an inhibitor of myosin contraction; scale bar, 100 μm. Bar figures represent quantification of area of 3D spheroids. (**C**) Phase contrast microscopy of spheroid formation using ESCs, MSCs, and cancer cells in 3D conditions with or without cholesterol depletion (1 μM Mev, 1.5 mM MβCD), and F-actin disruption (Cytochalasin D, CD, 1 μM). Scale bar, 100 µm. Bar figures represent quantification of area of 3D spheroids. (**D**) Immunofluorescence staining of polymerized F-actin (red fluorescence, rhodamine phalloidin) in spheroids formed by ESCs, MSCs, and cancer cells cultured in 3D conditions with or without Mev (1 μM), MβCD (1.5 mM), and CD (1 μM); Cell nuclei visualized with 4′,6-diamidino-2-phenylindole (DAPI) staining; scale bar, 20 µm. Bar figures represent quantification of F-actin intensity. For statistics, above data are presented as mean ± SEM. *, *p* < 0.05, **, *p* < 0.01, ***, *p* < 0.001, and ****, *p* < 0.0001, compared to 3D control or between conditions as indicated by bar.

The cytoskeleton is modulated during alterations of cell culture dimensionality [39], and this fundamental and ubiquitous cellular component is physically linked to components of the cell membrane including cholesterol, which is an integral part of the cell membrane [40,41,42].To assess whether interactions exist between the cholesterol pathway and cytoskeletal components during 3D spheroid formation of diverse cell types, we performed IPA which suggested that the cholesterol pathway may regulate 3D spheroid formation through F-actin polymerization as evidenced by interactions through RhoA and RAC1, two critical GTPases controlling the cytoskeleton (Figure 4A). To verify the predicted interactions between the cholesterol pathway and cytoskeletal elements, we disrupted myosin contraction and F-actin polymerization in 3D-cultured cells with blebbistatin or cytochalasin D, respectively, and we found that treatment with either drug significantly reduced 3D spheroid formation capacity across all cell types (Figure 4B,C). We also found that inhibition of cholesterol synthesis or depletion of cholesterol significantly enhanced 3D spheroid formation, which could be reduced by co-treatment with blebbistatin or cytochalasin D (Figure 4B,C). Furthermore, immunofluorescent staining demonstrated that inhibition of cholesterol synthesis or depletion of cholesterol in 3D conditions can increase polymerized F-actin as visualized by phalloidin staining, while treatment with cytochalasin D resulted in loss of polymerized F-actin (Figure 4D). Our findings demonstrate that cholesterol depletion induces 3D spheroid formation across diverse cell types via myosin contraction and F-actin stabilization.

### 3.5. Inhibition or Depletion of Cholesterol Increased 3D Spheroid Formation in Diverse Cell Types and Further Enhanced Cell-Specific Functional Outcomes

**Figure 5:**
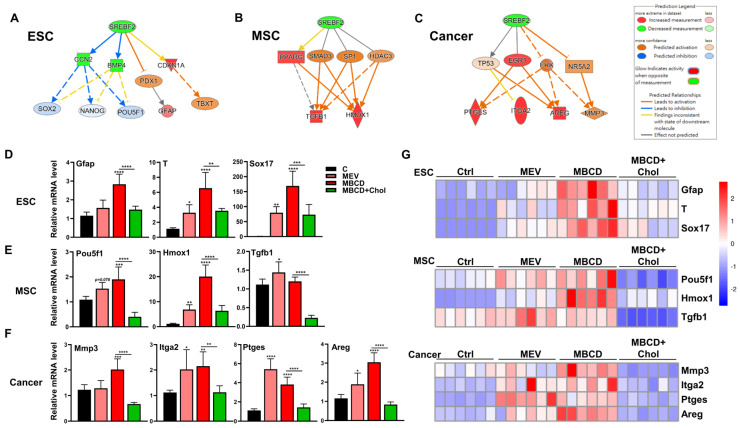
Inhibition or depletion of cholesterol increased 3D spheroid formation in diverse cell types and further enhanced cell-specific functional outcomes. IPA-based analysis for the regulation of sterol regulatory element binding transcription factor 2 (SREBF2) of 3D vs. 2D transcriptome profiles of (**A**) ESCs, (**B**) MSCs, and (**C**) cancer cells. (**D**) qPCR analysis for three germ layer genes (ectoderm: Gfap; Mesoderm: Tbxt/T; and Endoderm: Sox17) on ESCs cultured in 3D conditions with Mev (1 μM), MβCD (1.5 mM), or 1.5 mM MβCD + 100 μg/mL cholesterol treatment. (**E**) qPCR analysis for gene expression levels of pluripotency genes (Pou5f1) and immunomodulatory genes (Hmox1 and Tgfb1) in MSCs cultured in 3D conditions with Mev (1 μM), MβCD (1.5 mM), or 1.5 mM MβCD + 100 μg/mL cholesterol treatment. (**F**) qPCR analysis for gene expression levels of ECM modification enzyme (Mmp3), integrin molecule (Itga2), and inflammatory response factors (Ptges and Areg) in cancer cells cultured in 3D conditions with Mev (1 μM), MβCD (1.5 mM), or 1.5 mM MβCD + 100 μg/mL cholesterol treatment. Gapdh, internal control. qPCR data are presented as mean ± SEM. For statistics, above data are presented as mean ± SEM. *, *p* < 0.05, **, *p* < 0.01, ***, *p* < 0.001, and ****, *p* < 0.0001, compared to 3D control or between conditions as indicated by bar. (**G**) Heatmaps showing normalized expression levels of genes relevant to ESCs (**D**), MSCs (**E**), and cancer (**F**), illustrating the specific biological trends of each cell type following cholesterol modification during the process of spheroid formation.

To assess whether the increased 3D spheroid formation in diverse cells mediated by cholesterol inhibition has functional relevance, we performed IPA using the 2D and 3D transcriptome profiles to predict possible outcomes. IPA analysis indicated that downregulation of cholesterol synthesis promoted differentiation in ESCs (Figure 5A), higher stemness and immune modulation in MSCs (Figure 5B), and increased ECM modulation, integrin, and inflammation in cancer cells (Figure 5C). Functional validation by modulating the cholesterol pathway was performed on 3D culture of all three cell types: for ESCs, cholesterol inhibition with mevastatin or MβCD dramatically upregulated all three-germ layer-associated genes including Gfap, T, and Sox17; these trends were all reversed when exogenous cholesterol was added (Figure 5D). Cholesterol inhibition during 3D culture of MSCs elevated levels of Pou5f1, as well as the immunomodulatory genes Hmox1 and Tgfb1; these trends in all genes were consistently reversed when exogenous cholesterol was added (Figure 5E). For cancer cells, cholesterol inhibition during 3D culture increased levels of ECM modification enzyme Mmp3, the integrin Itga2, and inflammation response factors Ptges and Areg; these trends in all genes were consistently reversed when exogenous cholesterol was added (Figure 5F). To better visualize overall trends in all cell types, we summarized the gene expression results as heatmaps, and the results demonstrate that inhibition of cholesterol synthesis during 3D spheroid formation of all cell types further enhanced biological function specific for each cell type (Figure 5G). Taken together, cholesterol inhibition during the formation of 3D spheroid in diverse cell types further increased functional switching specific to each cell type, and addition of cholesterol resulted in reversal of functional switching.

### 3.6. Inhibition or Depletion of Cholesterol during 3D Spheroid Formation Increased Resistance to Oxidative Stress for All Cell Types

**Figure 6:**
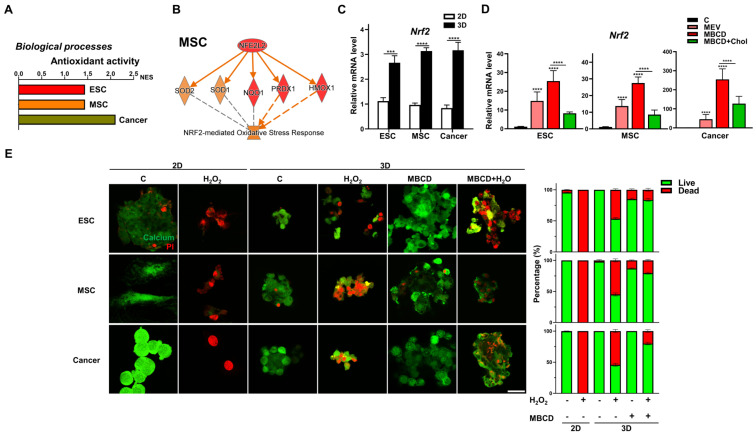
Inhibition or depletion of cholesterol during 3D spheroid formation increased resistance to oxidative stress for all cell types. (**A**) Significant enrichment and upregulation of the antioxidant activity gene sets via GSEA in ESCs, MSCs, and cancer cells cultured in 3D vs. 2D conditions. (**B**) IPA-based pathway analysis for the nuclear factor erythroid 2-related factor 2 (Nrf2; gene symbol Nfe2l2)-mediated antioxidant response of 3D- vs. 2D-cultured MSCs. Upregulated genes are indicated in red and downregulated genes in green. Solid and dashed lines indicate direct and indirect interactions, respectively. Results and individual gene expression levels for 3D- vs. 2D-cultured ESCs and cancer cells are shown in Figure S4. (**C**) qPCR analysis for expression levels of Nrf2 in ESCs, MSCs, and cancer cells cultured in 2D and 3D conditions; Gapdh, internal control. Data are presented as mean ± SEM. ***, *p* < 0.001, and ****, *p* < 0.0001, compared with 2D cultured conditions. (**D**) qPCR analysis for expression levels of Nrf2 in ESCs, MSCs, and cancer cells cultured in 3D conditions with Mev (1 μM), MβCD (1.5 mM), or 1.5 mM MβCD + 100 μg/mL cholesterol treatment; Gapdh, internal control. Data are presented as mean ± SEM. ****, *p* < 0.0001, compared to 3D control or between conditions as indicated by bar. (**E**) Live (green signal)/dead (red signal) staining of 2D-and 3D-cultured cells with or without hydrogen peroxide (H_2_O_2_; 500 nM for 2 h) and/or MβCD (1.5 mM) treatment. Scale bar, 25 μm. Bar figures represent quantification of the live and dead cell percentage in each condition.

Our bioinformatics analyses of these three cell types also revealed that 2D to 3D culture uniformly enriched and upregulated pathways related to antioxidant activity (Figure 6A). Pathway analysis revealed that the key transcription factor involved as nuclear factor erythroid 2-related factor 2 (Nrf2, encode by Nfe2l2) [43], and IPA further demonstrated in all three cell types after 3D spheroid formation, gene-network with Nrf2-mediated oxidative response-associated genes were consistently increased (Figure 6B and Figure S4). Validation with qPCR analyses demonstrated that 3D spheroid formation in all three cell types significantly upregulated Nrf2 (Figure 6C). Interestingly, inhibition of cholesterol synthesis during 3D spheroid formation increased Nrf2 expression in all cell types, whereas addition of cholesterol consistently decreased expression levels (Figure 6D). To validate these transcriptomic findings of increased resistance to oxidative stress in 3D spheroids of diverse cell types and cholesterol inhibition, we treated all cell types to exogeneous H_2_O_2_ to generate ROS, which in standard 2D culture resulted in profound cell death (Figure 6E). Interestingly, 3D spheroid formation compared to 2D conditions conferred resistance to exogenous H_2_O_2_-mediated ROS in all cell types, with increased viability clearly apparent. Impressively, cholesterol depletion during 3D spheroid formation further increased the proportion of viable cells as well as maintained better spheroid morphology as expected in all cell types (Figure 6E). Hence, resistance to oxidative stress is enhanced with 3D spheroid formation for all cell types and can be further improved with inhibition of cholesterol synthesis.

### 3.7. Inhibition or Depletion of Cholesterol Also Enhanced 3D Spheroid Formation across Diverse Human Cell Types

**Figure 7:**
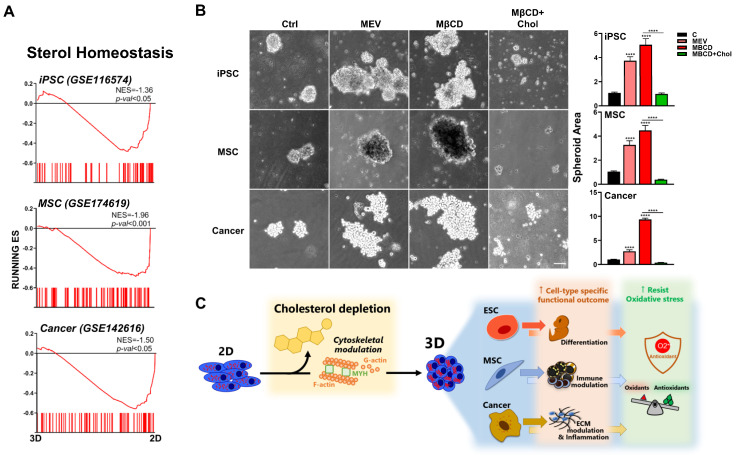
Inhibition or depletion of cholesterol also enhances 3D spheroid formation across diverse human cell types. (**A**) Bioinformatics analyses of public databases from each human cell type (GSE116574 for iPSCs, GSE174619 for MSCs, and GSE142616 for A549 lung adenocarcinoma cell line) were by GSEA. Significant enrichment and downregulation of the cholesterol synthesis pathway genes in human iPSCs, MSCs, and A549 cultured in 3D vs. 2D conditions. (**B**) Phase contrast microscopy of human iPSCs, MSCs, and SK-MES-1 lung squamous cancer cell line cultured in 3D conditions with Mev (1 μM), MβCD (1.5 mM), or 1.5 mM MβCD + 100 μg/mL cholesterol treatment. Scale bar, 100 μm. Bar figures represent quantification of area of 3D spheroids. Data are presented as mean ± SEM. ****, *p* < 0.0001, compared to 3D control or between conditions as indicated by bar (**C**). Summary diagram of results: downregulation of cholesterol synthesis pathway with cytoskeletal modulation is uniformly involved in 3D spheroid formation of diverse cell types, amplifying the specific biological potential of each cell type, as well as further enhancing 3D spheroid-conferred antioxidant ability for all cell types.

To assess whether our findings are relevant for human cells, since differences exist between murine and human cells even when isolated from similar organs/tissues, we first perform bioinformatics using transcriptome files of human cells which were cultured in 2D and 3D conditions: human iPSCs (GSE116574), P-MSCs (GSE174619), and lung adenocarcinoma cell line (A549; GSE142616). GSEA demonstrated significant enrichment and downregulation of cholesterol synthesis pathways in all three human cell types after 3D culture (Figure 7A). We then performed functional validation using these human stem cell and somatic cell types and found that either inhibition of cholesterol synthesis or depletion of cholesterol can increase 3D spheroid formation in human iPSCs, P-MSCs, and lung squamous cells (Figure 7B). Similar to that seen with the three murine cell types, addition of exogenous cholesterol (MβCD + cholesterol) decreased 3D spheroid formation in all evaluated human cell types; moreover, this process was seen for breast cancer cells (Figure S5) as well as lung cancer cells. Our results therefore demonstrate that 3D spheroid formation in diverse murine and human stem cell as well as somatic cell types result in distinct biologically relevant changes, which unexpectedly can be uniformly modulated through perturbation of the cholesterol pathway (Figure 7C). Moreover, modulation of the cholesterol pathway can also further enhance 3D spheroid formation-mediated resistance to oxidative stress. These findings collectively highlight 3D culture conditions as being more physiologically relevant, as well as demonstrating the importance of the cholesterol pathway in modulation of cell-specific biological function and ROS resistance.

## 4. Discussion

Recent advances in in vitro culture technology have demonstrated that spheroid formation in 3D conditions likely provides more physiologically relevant information than classical 2D conditions [10,11,44]. We chose to use the multicellular 3D spheroid culture method because it is one that is most commonly utilized, including non-cancer cell types (i.e., ESCs and MSCs) [9,10] as well as cancer cell types. In pluripotent stem cells, 3D spheroid formation—also known as embryoid body formation—represents loss of stemness/pluripotency [8,9], while in somatic stem cells, 3D spheroid formation represents stemness and immunomodulatory function [35,36]. Three-dimensional cancer spheroids have been found to better model disease progression—i.e., ECM modification and inflammatory microenvironment—than the standard 2D culture [13,45,46]. While multiple cell types are capable of spheroid formation in 3D culture systems, surprisingly little is known about uniformed pathways involved in this process in diverse cell types. Using comprehensive microarray analyses with subsequent functional validation of 3D- vs. 2D-cultured mouse ESCs, MSCs, and cancer cells, we found that 3D spheroid formation promotes biological processes specific for each cell type. Uniformed processes found across all cell types after 3D spheroid formation include downregulation of the cell cycle [13,31,32,33], which we found as well; this could reflect the ability of 3D culture to mimic the natural quiescent state of adult stem cells and somatic cells [47]. Unexpectedly, we found that 3D spheroid formation led to downregulation of cholesterol synthesis in all cell types tested, which involved myosin contraction and cytoskeletal F-actin stabilization. Perturbations of this pathway not only enhanced 3D spheroid formation in all cell types tested but also altered the specific biological processes of each cell type and additionally further increased resistance to oxidative stress already conferred by 3D spheroid formation. These results were consistent not only across diverse cell types but across murine as well as human cells; moreover, in the human system, results were consistent between two types of lung cancer cells (adenocarcinoma and squamous cell cancer) and breast cancer cells. Our findings, therefore, not only provided new mechanistic insights into uniformed biological pathways involved in 3D formation of diverse cell types but also offer a possible method to enhance ex vivo cell viability, which can have strong translational implications, since cell therapy requires ex vivo cell expansion to achieve the high cell volumes necessary for clinical application.

The finding of downregulation of the cholesterol synthesis pathway during 3D spheroid formation of diverse murine and human cell types was unexpected and intriguing. Medications modulating this pathway such as statins are among the most commonly used agents worldwide for control of hyperlipidemia and cardiovascular disease risk. However, there have been no reports on cholesterol modulation for in vitro 3D spheroid formation. The only existing literature on in vitro use of statins is for treatment of standard 2D-cultured cancer cells, but the doses used in such studies—most commonly at 10 μM—are at least 10 times higher than circulating levels found in patients, and therefore are not clinically relevant [48]. In our study, we utilized a dose of 1 μM which was sufficient to enhance 3D spheroid formation without inducing significant cytotoxicity. In fact, in a number of large epidemiological studies and randomized controlled studies, statin use and low cholesterol levels are associated with increased cancer risk and mortality [49,50]. Given that 3D culture and spheroid formation are often used to select for cancer-initiating cells [12,13], our data in conjunction with epidemiological/clinical data may point to a role for cholesterol in cancer progression which was not uncovered in previous in vitro studies using 2D systems and high statin doses. While there are currently no reports on the influence of the cholesterol pathway on 3D spheroid formation of normal, non-cancerous stem cells, in the few reports using standard 2D culture, it does appear that perturbations of this pathway can affect the stemness/differentiation capacity of both adult stem cells as well as pluripotent stem cells [51,52,53]. Our data demonstrates the importance of the cholesterol synthesis pathway in modulating cell-specific fates as well as 3D spheroid formation capacity.

We also found that 3D spheroid formation increases cellular resistance to oxidative stress in all cell types, which can be further enhanced with inhibition of the cholesterol synthesis pathway. While few reports have directly assessed whether 3D spheroid formation increases antioxidant capacity, antioxidant treatment has been shown to improve 3D-cultured cell viability exposed to ROS stress [19,54,55]. Our bioinformation analyses and validation data suggest that upregulation of Nrf2 is involved in the increased resistance to oxidative stress of 3D spheroids as well as with cholesterol depletion. In cancer 3D spheroids, activation of the Nrf2 pathway plays a role in increasing cell survival [56], and in two studies using 2D-cultured non-cancer cells, direct addition of cholesterol led to increased cellular ROS [57] with activation of the Nrf2 pathway as a defense mechanism to prevent additional harm [58]. Moreover, in a number of rodent studies, inhibition of cholesterol synthesis with statins can enhance antioxidative capacity, partially through Nrf2 [59]. Most recently, in a rat model of high-fat diet-induced renal disease, statins were found to attenuate cholesterol-induced ROS [60]. The ability of our data utilizing bioinformatics analyses and functional validation of 3D spheroids of diverse cell types to capture such information strongly implicates the better capacity of 3D cultured cells to reflect in vivo processes as well as provide translationally relevant information.

One strength of our study is in comprehensively assessing diverse cell types—including primary stem cells and cancer cell lines—across two species. We also found 3D culture conditions, which can allow for cell therapy application requiring scale-up to achieve large cell volumes, as well as high-throughput drug testing. The profound changes in cholesterol transport, cytoskeletal elements, and cell cycle dynamics dimensionality that our study revealed in numerous types of mammalian cells strongly implicate that simplistic, reductionist information yielded by the classical 2D in vitro culture system is likely less relevant to in vivo systems. Moreover, for cancer biology, there are numerous methods of 3D culture, with each method appearing to highlight a different aspect of the complex disease process [13]. In fact, a number of recent publications demonstrate the versatility of 3D culture systems in cancer research, including introducing additional cell types and engineering techniques [61,62,63]. Importantly, we found that cholesterol modulation during 3D spheroid formation regulated the specialized biological potential of each cell type. In a very recent study, the addition of cancer-associated fibroblasts appears to secrete cholesterol which stabilized 3D cancer spheroids [64]. Our findings therefore contribute to the increasing consensus on the more physiologically relevant 3D in vitro culture systems and the potential of therapy application [45,64].

## 5. Conclusions

In summary, through bioinformatics analyses with functional validation of 2D- and 3D-cultured diverse cell types, we demonstrate the downregulation of cell cycle dynamics and cell proliferation as well as cholesterol synthesis and upregulation of antioxidant capacity are uniformed pathways involved in 3D spheroid formation of diverse cell types, including pluripotent stem cells, somatic cells, and cancer cells. Specifically, either decreased synthesis or depletion of cholesterol enhances 3D spheroid formation across diverse murine and human cell types, with involvement of myosin contraction and F-actin polymerization. Cholesterol inhibition during 3D spheroid formation further enhanced the biological potential specific for each cell type as well as antioxidant capacity. Our findings demonstrate new unified mechanisms involved in in vitro 3D spheroid formation across diverse murine and human cell types that can enhance functional outcomes as well as resistance to oxidative stress, both of which can help further translational application.

## Data Availability

The microarray data generated in this study have been deposited to NCBI GEO database and the accession number is GSE99942. The other data that support the findings of this study are available from the corresponding author upon reasonable request.

## Supplementary Materials

The following supporting information can be downloaded at: https://www.mdpi.com/article/10.3390/cells12162050/s1.
