# Supplementary material for: Targeting Conserved Pathways in 3D Spheroid Formation of Diverse Cell Types for Translational Application: Enhanced Functional and Antioxidant Capacity
Source: Cells. 2023 Aug 11;12(16):2050. doi: 10.3390/cells12162050 (PMC10453086; doi:10.3390/cells12162050)
Supplement: Supplementary file 1 [file cells-12-02050-s001.zip › Sup information.pdf]

Supplemental Information

I. Supplemental figure legends

Figure S1: Related to Figure 1. Ingenuity Pathway Analysis (IPA)-based pathway analysis for cell cycle related genes of mouse ESCs and lung cancer cells cultured in 3D vs. 2D conditions. IPA-based analysis for cell cycle related genes of (A) mouse ESCs and (B) lung cancer cells cultured in 3D vs. 2D conditions. Upregulated genes are indicated in red and downregulated genes in green. Solid and dashed lines indicate direct and indirect interactions, respectively. Genes in white circles generated by based on IPA and not from transcriptome data.

Figure S2: Related to Figure 3. IPA-based analysis for the cholesterol synthesis related genes of mouse ESCs and lung cancer cells cultured in 3D vs. 2D conditions. IPA-based analysis for the cholesterol synthesis related genes of (A) mouse ESCs and (B) lung cancer cells cultured in 3D vs. 2D conditions. Upregulated genes indicated in red and downregulated genes in green. Solid and dashed lines indicate direct and indirect interactions, respectively. Genes in white circles generated by based on IPA and not from transcriptome data.

Figure S3: Related to Figure 3. Cell viability assessment for mevastatin. (A) Dose-dependent viability assessment of lung cancer cells cultured in 2D conditions at 24 hours as determined by the 3-4,5-dimethylthiazol-2-yl)-2,5-diphenyltetrazolium bromide (MTT) assay. (B) qPCR analysis for expression levels of major cholesterol synthesis enzymes *Hmgcr*, *Dhcr24*, *Msmo1*, and *Sqle* after 1μM mevastatin treatment for 24 hours in 2D-cultured lung cancer cells; *Gapdh*, internal control. Data are presented as mean ± SEM. *, $p < 0.05$, **, $p < 0.01$, and ***, $p < 0.001$, compared with control.

Figure S4: Related to Figure 6. IPA-based analysis for Nrf2-mediated antioxidant response of mouse ESCs and lung cancer cells cultured in 3D vs. 2D conditions. IPA-based analysis for the Nrf2-mediated antioxidant response related genes of (A) mouse ESCs and (B) lung cancer cells cultured in 3D vs. 2D conditions. Upregulated genes indicated in red and downregulated genes in green. Solid and dashed lines indicate direct and indirect interactions, respectively.

Figure S5: Related to Figure 7. Inhibition or depletion of cholesterol also

enhance 3D spheroid formation of human MCF breast cancer cell. Phase contrast microscopy of human MCF7 breast cancer cell line cultured in 3D conditions with Mev (1 μM), MβCD (1.5 mM) or 1.5 mM MβCD + 100 μg/ml cholesterol treatment. Scale bar, 100 μm. Bar figures represent quantification of area of 3D spheroids. Data are presented as mean ± SEM. ****, $p < 0.0001$, compared to 3D control or between conditions as indicated by bar.

II. Supplemental table legends

Table S1: List of cells, chemicals, reagents, software, and equipment resources used in the study.
Table S2: Detailed primer and target gene information.
