# Supplementary figures and images for: Targeting Conserved Pathways in 3D Spheroid Formation of Diverse Cell Types for Translational Application: Enhanced Functional and Antioxidant Capacity
Source: Cells. 2023 Aug 11;12(16):2050. doi: 10.3390/cells12162050 (PMC10453086; doi:10.3390/cells12162050)

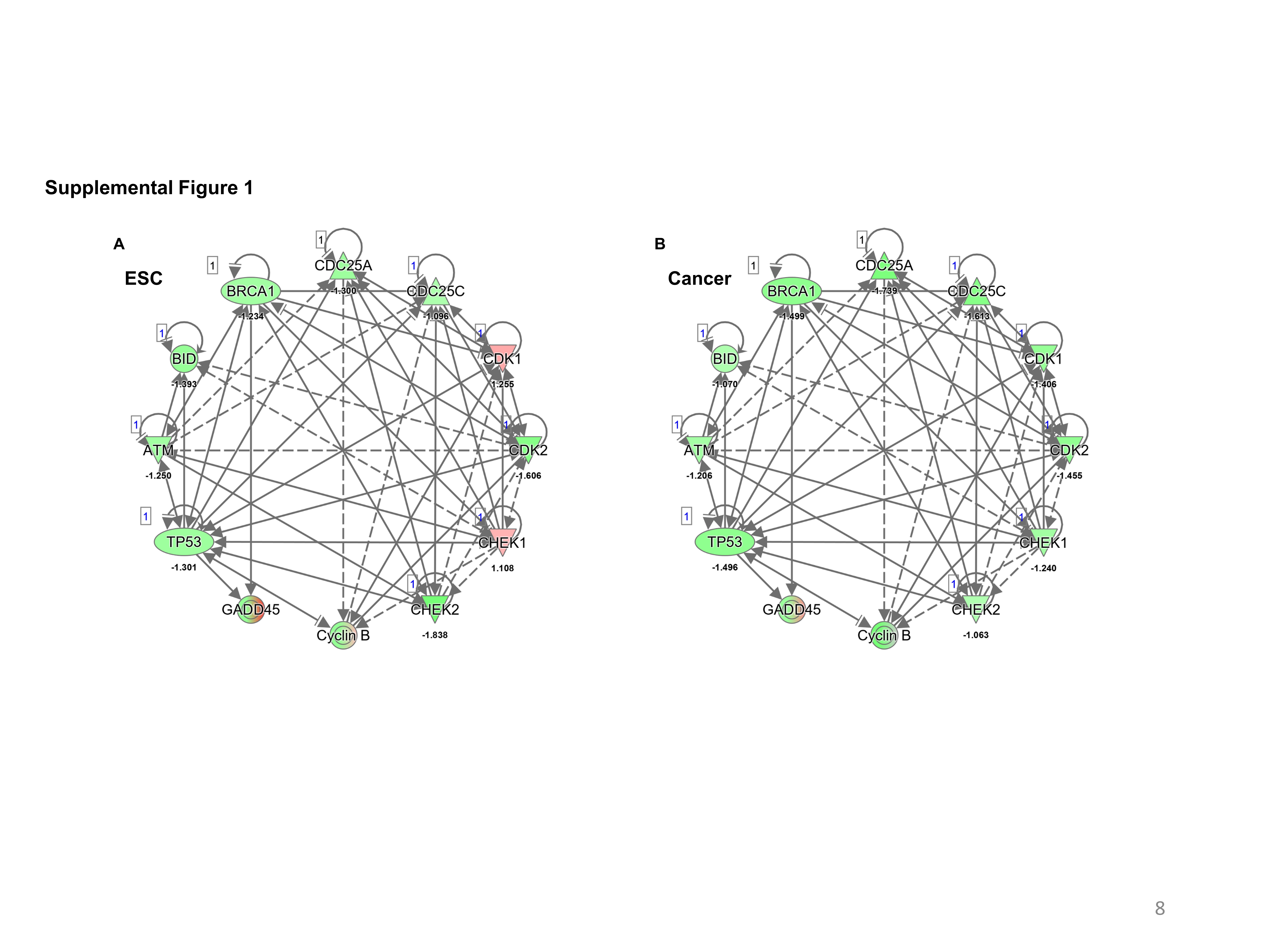

Supplement: Supplementary file 1 [file cells-12-02050-s001.zip › SupFig1.TIF]

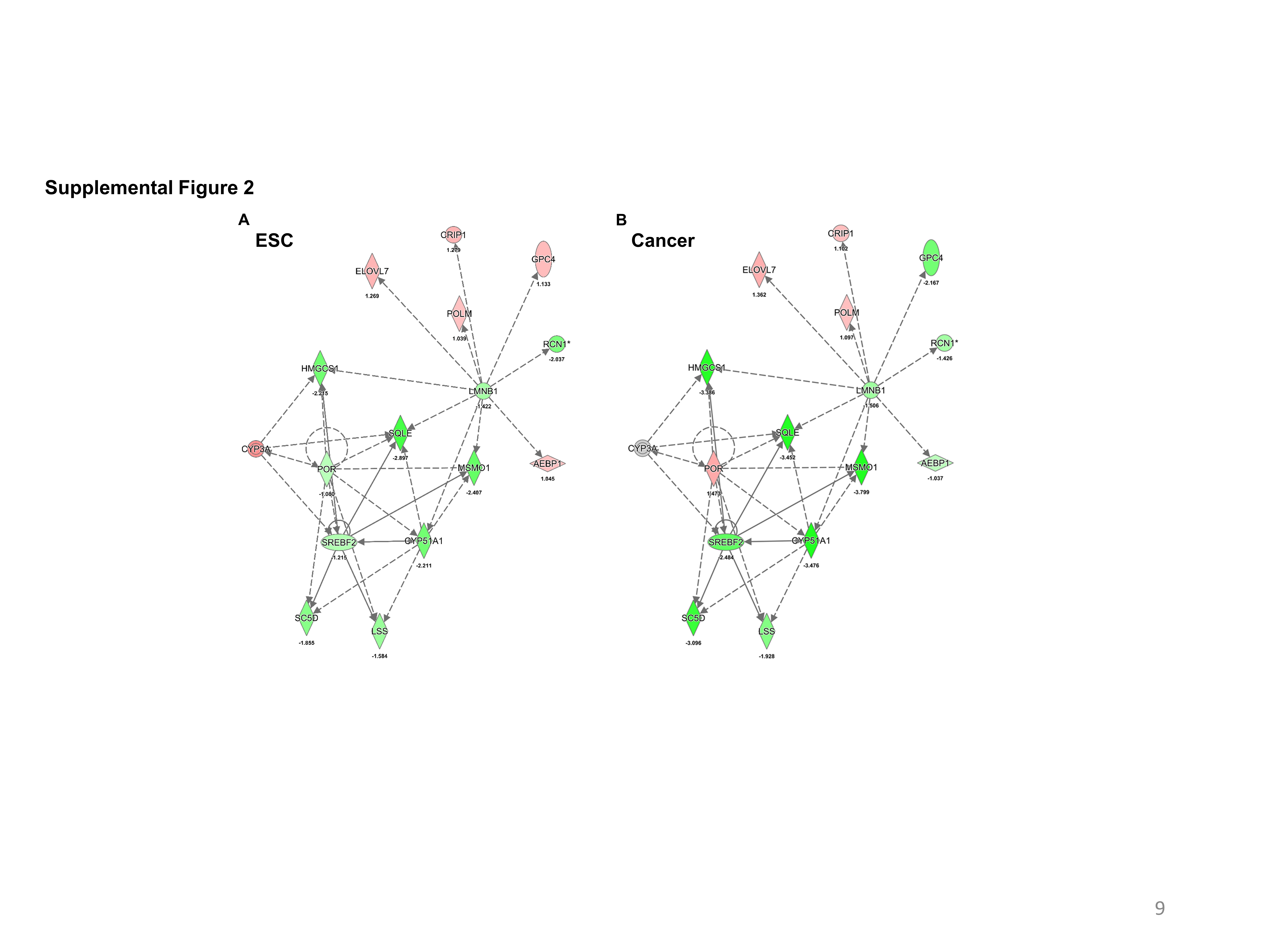

Supplement: Supplementary file 1 [file cells-12-02050-s001.zip › SupFig2.TIF]

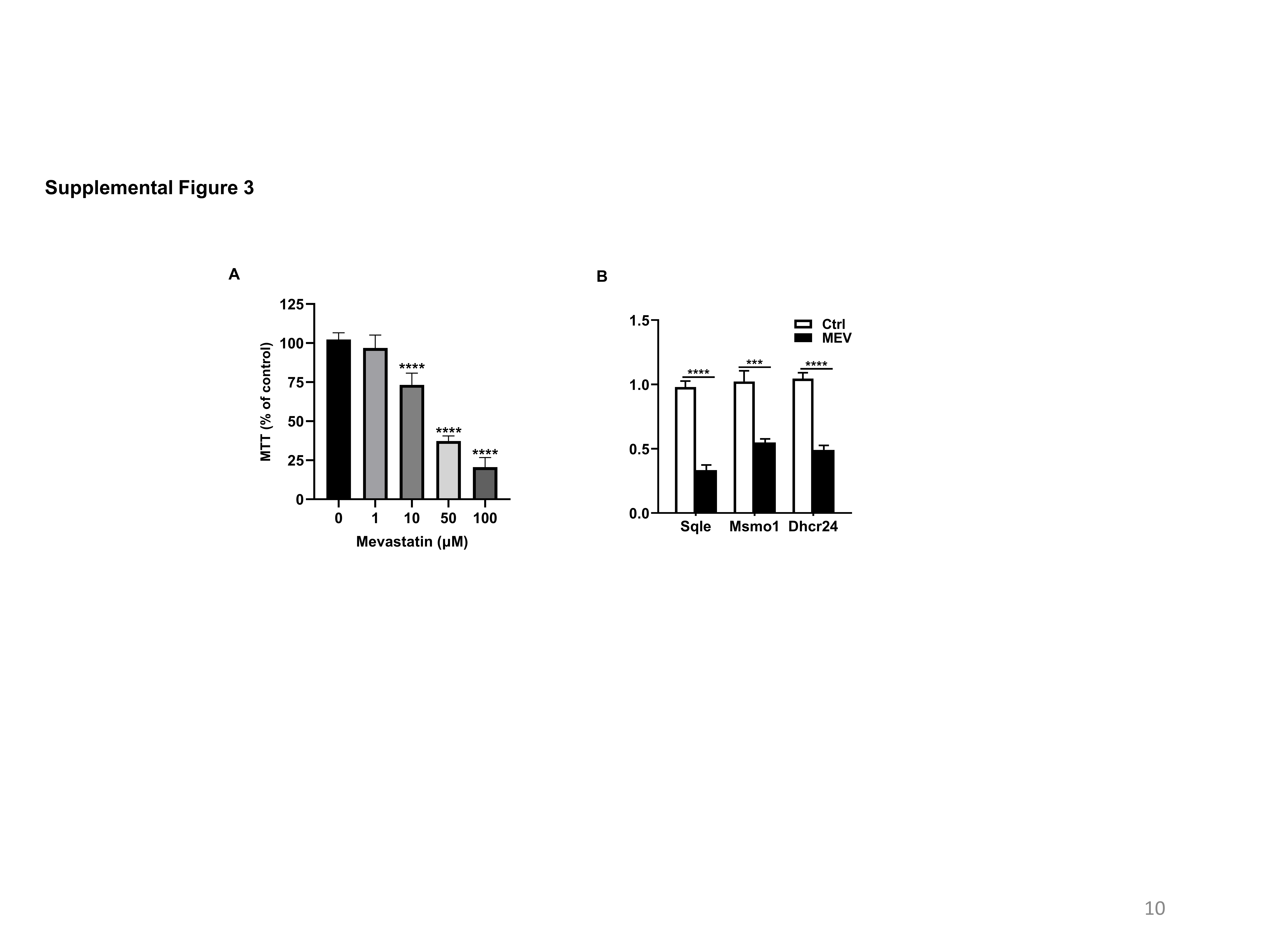

Supplement: Supplementary file 1 [file cells-12-02050-s001.zip › SupFig3.TIF]

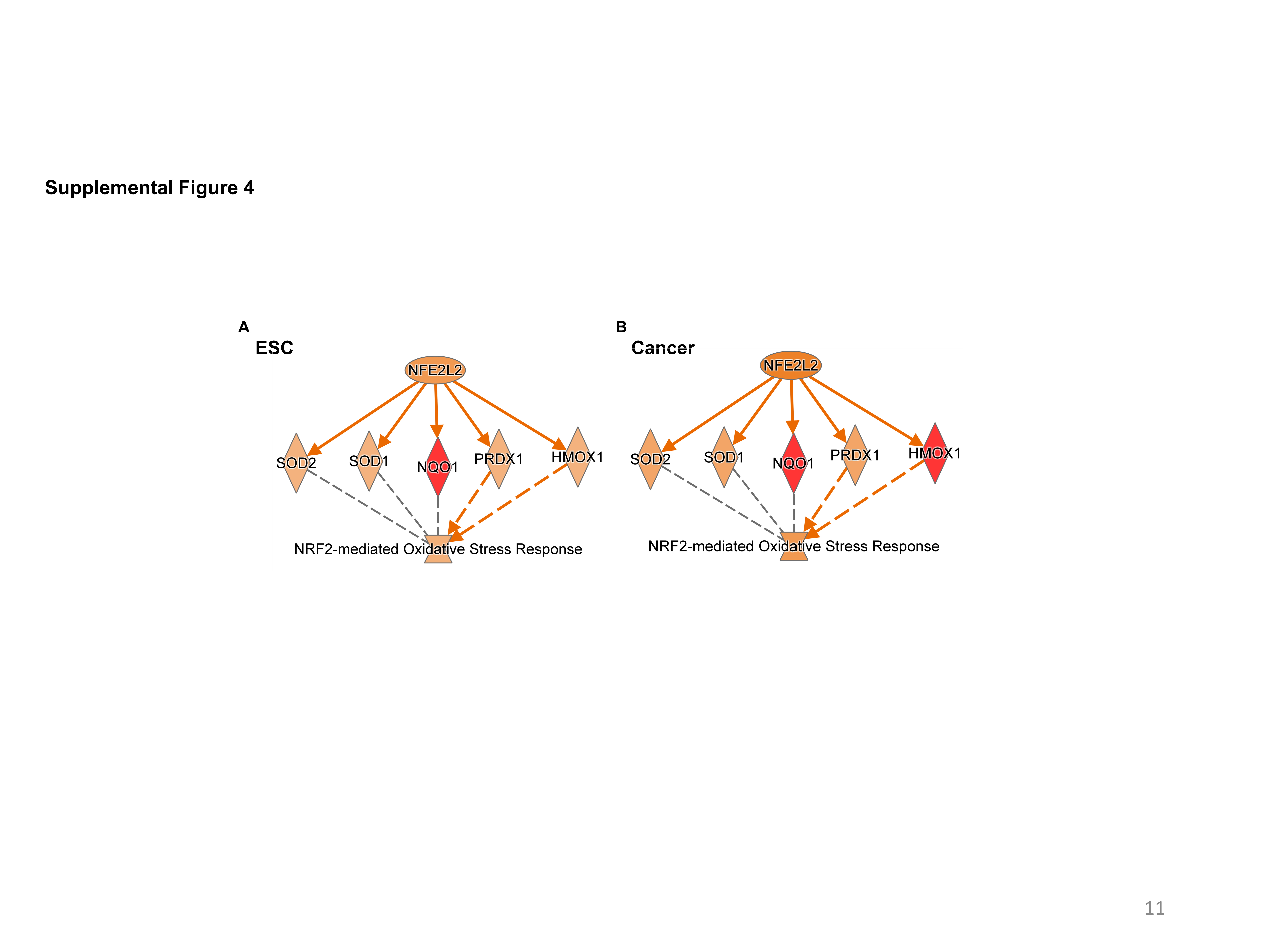

Supplement: Supplementary file 1 [file cells-12-02050-s001.zip › SupFig4.TIF]

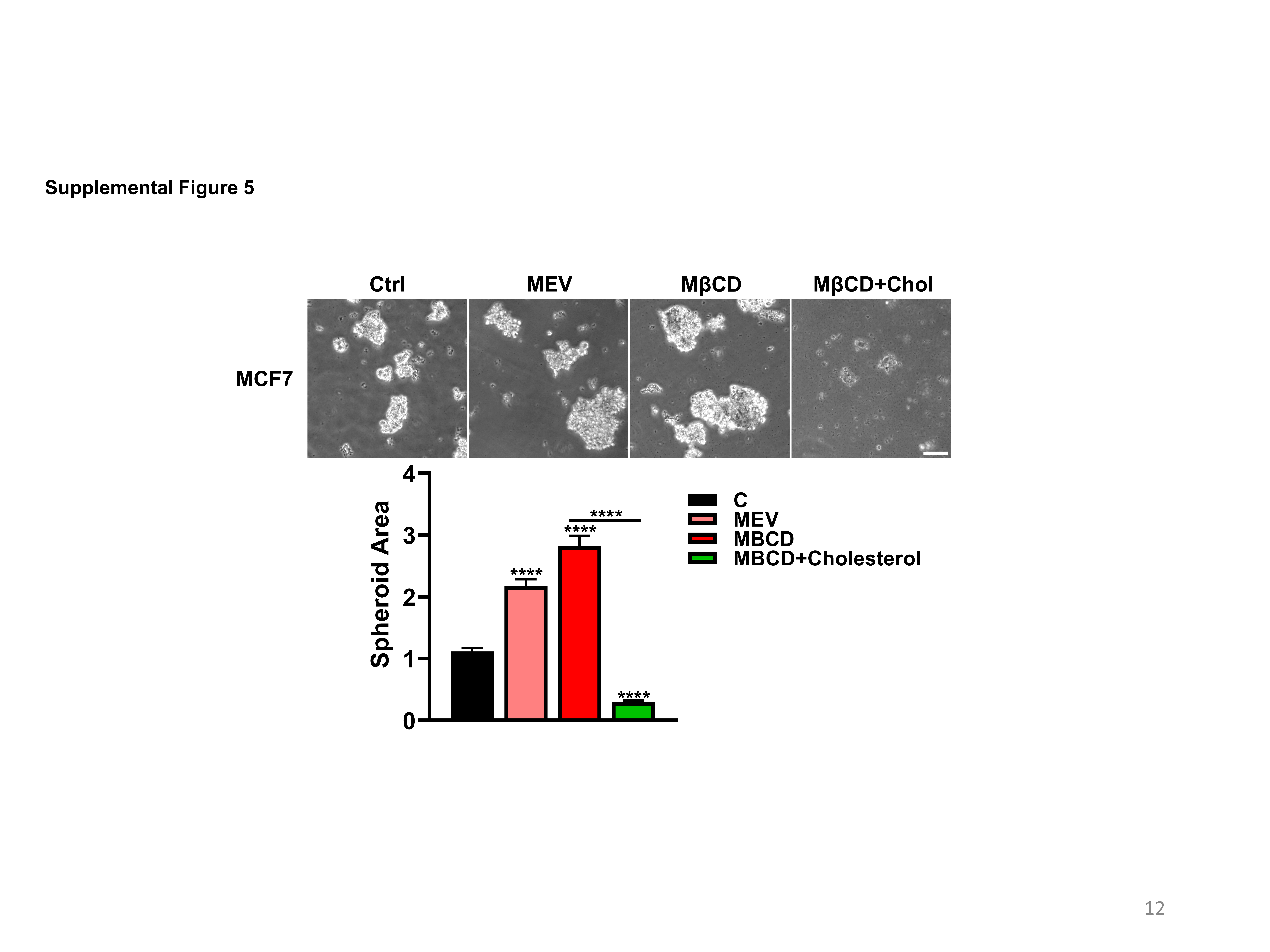

Supplement: Supplementary file 1 [file cells-12-02050-s001.zip › SupFig5.TIF]
